# Gallbladder ascariasis in Kosovo – focus on ultrasound and conservative therapy: a case series

**DOI:** 10.1186/s13256-017-1536-4

**Published:** 2018-01-13

**Authors:** Vlora Ismaili-Jaha, Halim Toro, Lidvana Spahiu, Mehmedali Azemi, Teuta Hoxha-Kamberi, Muharrem Avdiu, Shqipe Spahiu-Konjusha, Luan Jaha

**Affiliations:** 1 Department of Pediatric Gastroenterology, University Clinical Center of Kosovo, Prishtina, Kosovo; 2 Department of Pediatric Nephrology, University Clinical Center of Kosovo, Prishtina, Kosovo; 3 Department of Pediatric Genetics, University Clinical Center of Kosovo, Prishtina, Kosovo; 4 Faculty of Nursing, University College AAB Prishtina, Prishtina, Kosovo

**Keywords:** Gallbladder, Ascariasis, Clinical features, Ultrasonography, Treatment

## Abstract

**Background:**

*Ascaris lumbricoides* is one of the most common intestinal infections in developing countries, including Kosovo. In contrast to migration to the bile duct, migration of the worm to the gallbladder, due to the narrow and tortuous nature of the cystic duct, is rare. When it does occur, it incites acalculous cholecystitis.

**Case presentations:**

This case series describes a 16-month-old Albanian girl, a 22-month-old Albanian girl, a 4-year-old Albanian girl, and a 10-year-old Albanian boy. Here we report our experience with gallbladder ascariasis including clinical manifestations, diagnostic procedures, and treatment. Fever, diarrhea and vomiting, dehydration, pale appearance, and weakness were the manifestations of the primary disease. In all patients, a physical examination revealed reduced turgor and elasticity of the skin. Abdomen was at the level of the chest, soft, with minimal palpatory pain. The liver and spleen were not palpable. A laboratory examination was not specific except for eosinophilia. There were no pathogenic bacteria in coproculture but *Ascaris* was found in all patients. At an ultrasound examination in all cases we found single, long, linear echogenic structure without acoustic shadowing containing a central, longitudinal anechoic tube with characteristic movement within the gallbladder. Edema of the gallbladder wall was suggestive of associated inflammation. There were no other findings on adjacent structures and organs. All patients received mebendazole 100 mg twice a day for 3 days. They also received symptomatic therapy for gastroenteritis. Because of elevated markers of inflammation all patients were treated with antibiotics, assuming acute cholecystitis, although ultrasound was able to confirm cholecystitis in only two of our four patients. Since the length of stay was dependent on the primary pathology it was 7 to 10 days. At control ultrasounds on 14th day, third and sixth month, all patients were free of ascariasis.

**Conclusions:**

Gallbladder ascariasis should be considered in all patients presenting with abdominal pain, distension, colic, nausea, anorexia, and intermittent diarrhea associated with jaundice, nausea, vomiting, fever, and severe radiating pain. Eosinophilia, ova, and parasites on stool examination as well as an anechogenic tube with characteristic movement within the bile duct found on abdominal ultrasound are conclusive for diagnosis. Mebendazole is an effective drug for the treatment. Surgical treatment is rarely needed.

## Background

*Ascaris lumbricoides* is the most frequent human intestinal nematode. It is prevalent especially in most tropical and subtropical regions, but has worldwide distribution. It infects approximately 25% of the world’s population [1]; around 20,000 deaths occur per year from an adverse clinical course of the disease. In endemic areas, *Ascaris lumbricoides* accounts for 50 to 60% of pediatric admissions in surgical emergency departments. Hepatobiliary and pancreatic ascariasis accounts for approximately 10% of such admissions [2].

Although usually benign in prognosis, *Ascaris* is associated with a lot of complications, including hepatobiliary ascariasis, intestinal obstruction by the bolus of worms, pancreatic ascariasis, acute appendicitis, peritoneal granulomas, small bowel volvulus, and small bowel intussusception.

Statistics about the prevalence of ascariasis in Kosovo are deficient. However, it is a rare condition and in 2016 it was responsible for the admission of only four patients, or only 0.36% of all admissions at the Clinic of Pediatrics at the University Clinical Hospital in Kosovo that acts as a referral center for around 2 million people.

## Case presentations

Here we present four Albanian children with gallbladder ascariasis treated at our Department of Gastroenterology during the last year.

### Case 1

A 16-month-old Albanian girl presented at our Department with acute gastroenteritis. Fever, diarrhea, and vomiting were her main symptoms. She was dehydrated, pale, and weak. Her history of nutrition and immunization was normal for her age. A physical examination revealed reduced turgor and elasticity of skin, but no other findings. Her abdomen was at the level of her chest, soft, with minimal palpatory pain. Her liver and spleen were not palpable.

A laboratory examination found her erythrocyte sedimentation rate at level of 60 mm at the first hour, white blood cells (WBC) count 12.400, and C-reactive protein of 41 mg/L. There were 15% eosinophils of overall WBC count; glucose level of blood was 10.9 mmol/L. Glucose was positive in urine as well as acetone and proteins. Liver tests were normal.

*Ascaris* worm and ova were found in stool that was otherwise negative for bacteria, adenovirus, and rotavirus.

**Fig. 1:**
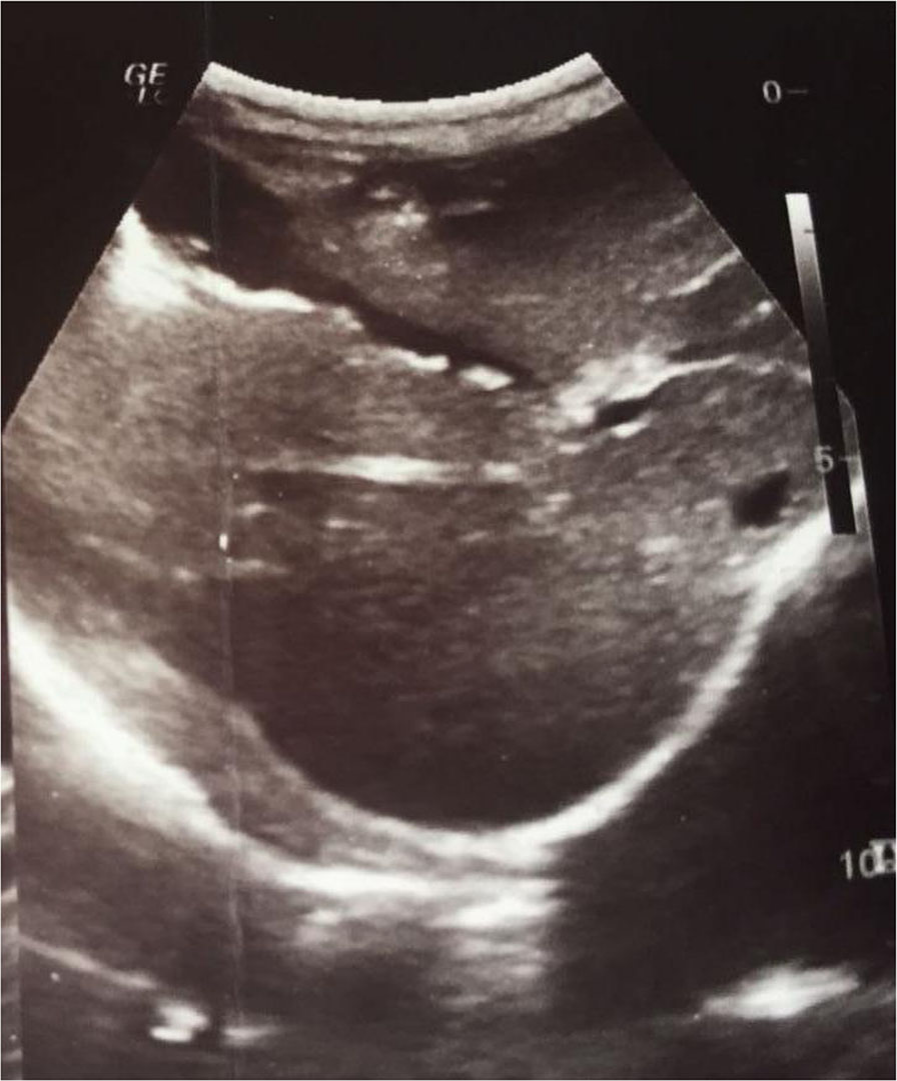
Ascaris worm in gallbladder

At abdominal ultrasound, we found a single, long, linear echogenic structure without acoustic shadowing containing a central, longitudinal anechoic tube with characteristic movement within the gallbladder. Edema of the gallbladder wall was suggestive of associated inflammation. There were no other findings on adjacent structures and organs (Fig. 1).

After the diagnosis was established, our patient was given antihelminthic therapy with mebendazole, 100 mg twice a day for 3 days and was free of the disease at the control visit. Because of elevated markers of inflammation and acute cholecystitis at ultrasound, she was treated with antibiotics.

### Case 2

A 22-month-old Albanian girl presented at our Department with acute gastroenteritis. Fever, diarrhea and vomiting, dehydration, and weakness were her main symptoms. A physical examination revealed reduced turgor and elasticity of skin, but was otherwise normal.

**Fig. 2:**
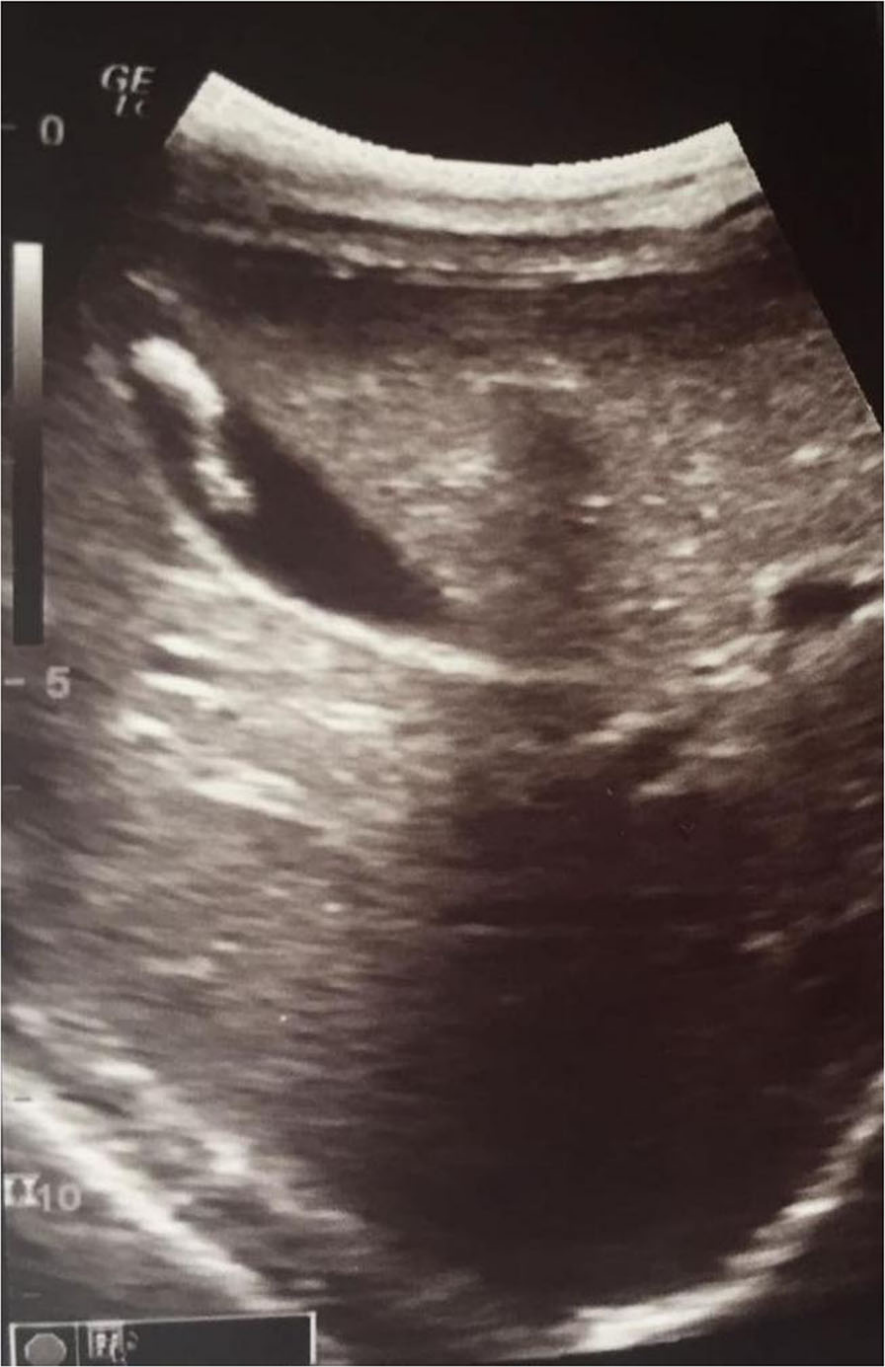
Ascaris worm movement

A laboratory examination found her erythrocyte sedimentation rate at a level of 36 mm at the first hour, WBC count 18.000, and C-reactive protein of 12 mg/L. There were 10% eosinophils of overall WBC count. Other tests were normal. *Ascaris* worm and ova were found in stool that was otherwise negative for bacteria, adenovirus, and rotavirus. An abdominal ultrasound found features characteristic for gallbladder ascariasis without associate inflammation (Fig. 2)

After the diagnosis was established, she was given antihelminthic therapy with mebendazole, 100 mg twice a day for 3 days, and antibiotics due to elevated markers of inflammation. At the control examination, she was free of disease.

### Case 3

A 4-year-old (50-month old) Albanian girl presented at our Department with fever, diarrhea, and vomiting due to acute gastroenteritis. She was mildly dehydrated. A physical examination was otherwise normal.

**Fig. 3:**
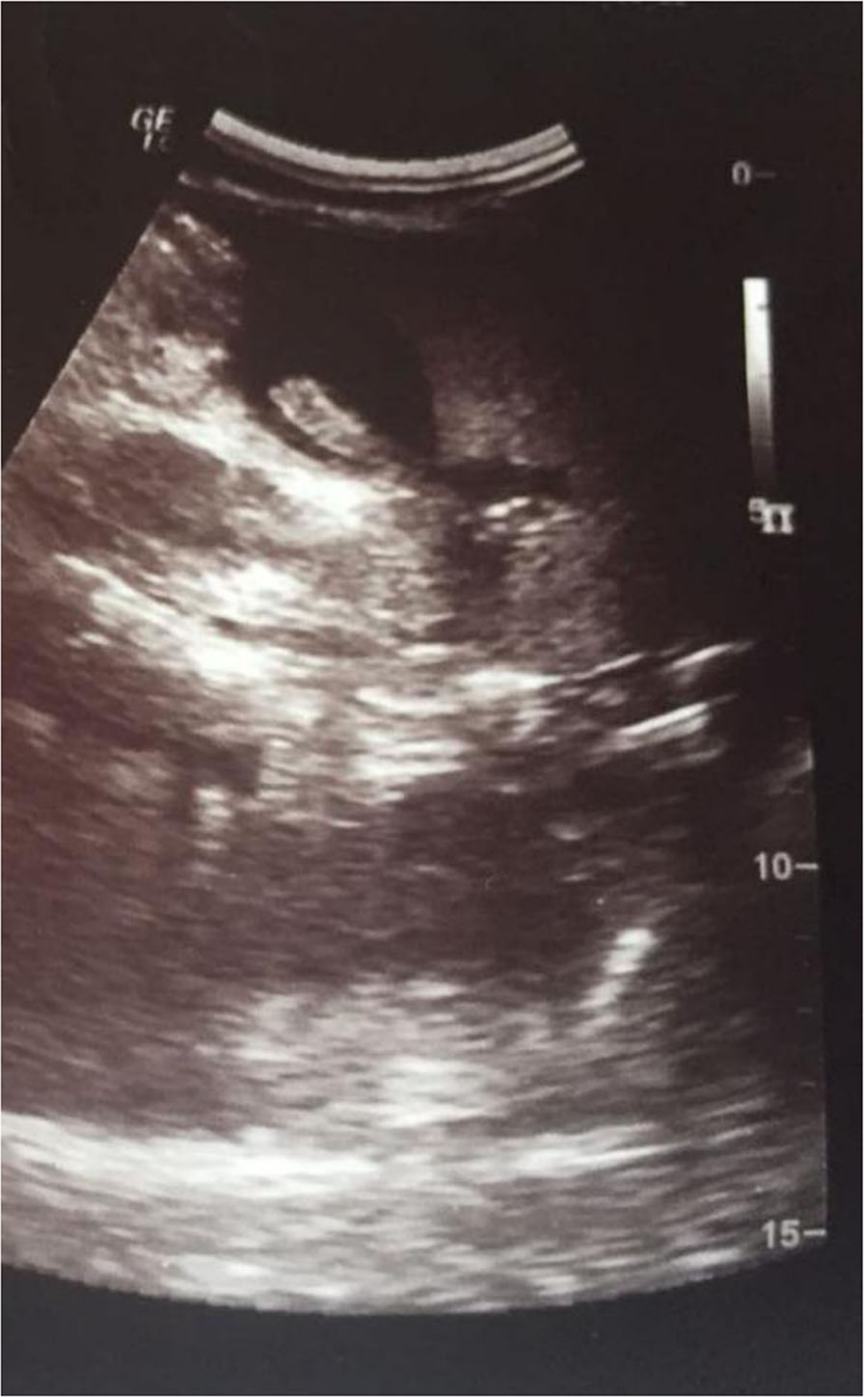
Ascaris worm in gallbladder

A laboratory examination found her erythrocyte sedimentation rate at a level of 40 mm at the first hour, WBC count 14.000, and C-reactive protein of 18 mg/L. There were 14% eosinophils of overall WBC count. Other tests were normal. *Ascaris* worm and ova were found in stool. An abdominal ultrasound found features characteristic for gallbladder ascariasis without associate inflammation (Fig. 3).

After the diagnosis was established, she was treated with mebendazole, 100 mg twice a day for 3 days. Due to elevated markers of inflammation an antibiotic was also given. At the control examination, she was free of disease.

### Case 4

A 10-year-old (130-month old) Albanian boy presented at our Department with mild fever, but numerous episodes of diarrhea and vomiting. He was severely dehydrated and weak. The admission diagnosis was acute gastroenteritis. A physical examination revealed reduced turgor and elasticity of skin and little pain during the abdominal palpation.

A laboratory examination found his erythrocyte sedimentation rate at a level of 30 mm at the first hour, WBC count 15.000, and C-reactive protein of 24 mg/L. There were 14% eosinophils of overall WBC count. Other tests were normal. *Ascaris* worm and ova were found in stool that was otherwise negative for bacteria, adenovirus, and rotavirus. An abdominal ultrasound found features characteristic for gallbladder ascariasis with associate inflammation.

**Fig. 4:**
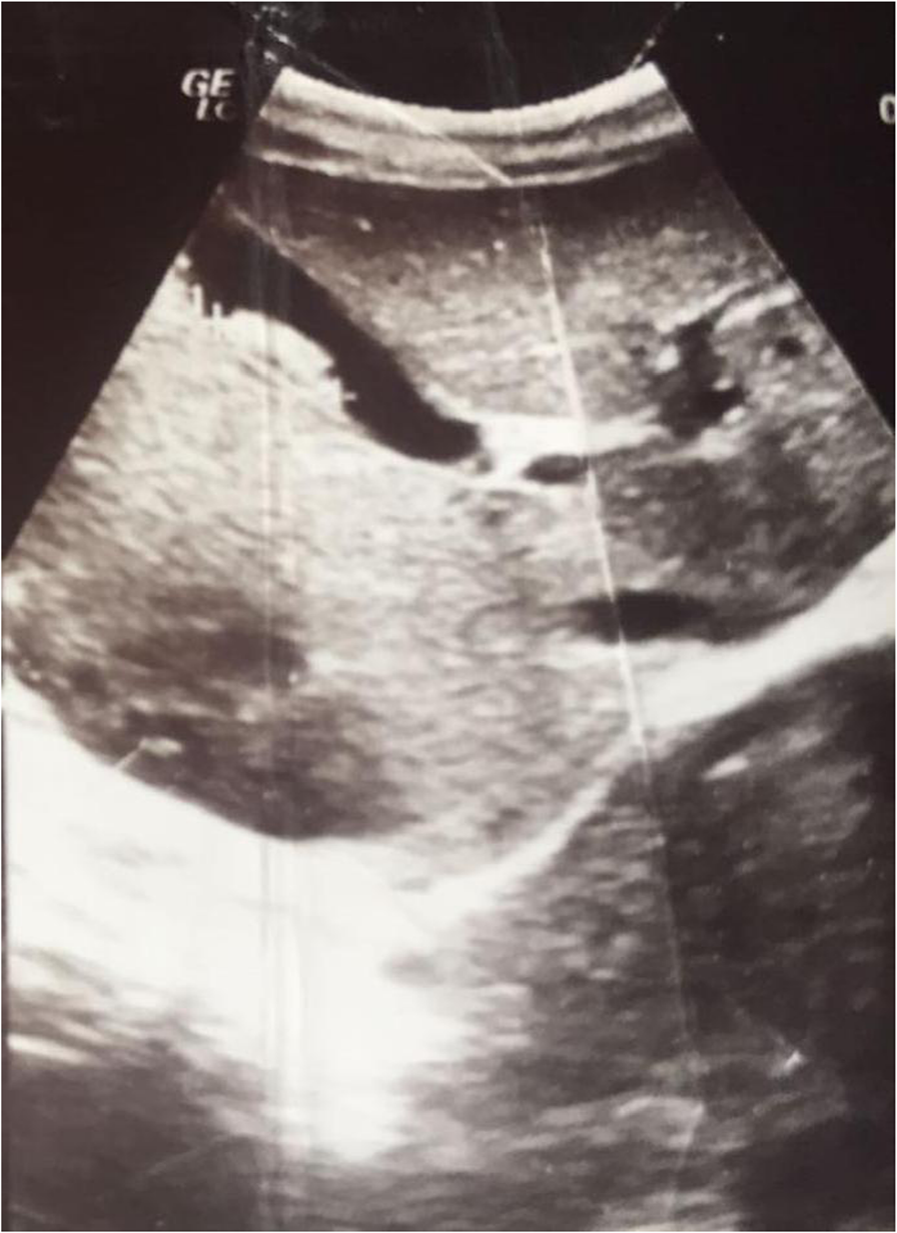
Ascaris worm in gallbladder

After the diagnosis was established, he was given antihelminthic therapy with mebendazole, 100 mg twice a day for 3 days, and antibiotics due to elevated markers of inflammation and characteristic feature of cholecystitis at ultrasound examination (Fig. 4).

## Discussion

Infestation of the gallbladder with *Ascaris* worms is rare [3, 4], because the narrow and tortuous cystic duct limits access to the gallbladder. Worms or their eggs in the common bile duct and cystic duct obstruct the gallbladder leading to its distension and acute cholecystitis. Worm load in the gut, worm size, sex, and age of patients are determining factors influencing migration of a parasite toward the gallbladder [5].

Abdominal pain, distension, colic, nausea, anorexia, and intermittent diarrhea may be manifestations of partial or complete intestinal obstruction by adult worms. If the patients develop cholangitis, pancreatitis, or appendicitis jaundice, then nausea, vomiting, fever, and severe radiating pain will be present.

A complete blood count showing eosinophilia may indicate helminthic infection. However, it is not specific and diagnosis depends on a stool examination for ova and parasites. Large, brown 60 × 50 μm three-layered eggs in patients are characteristic for ascariasis. Serological tests are not clinically useful for ascariasis.

When it comes to biliary ascariasis, abdominal sonography is key to diagnosis, even though sonographic findings might be confused by zigzag and meandering movements of active live worms [6]. The characteristic sonographic features of worms in the bile duct are the presence in the gallbladder of a single, long, linear or curved echogenic structure without acoustic shadowing, looking like an anechogenic tube, and characteristic movement of these long echogenic structures within the bile duct. Other features are less specific or are an expression of the complications and consist of gallbladder distention, edema of the gallbladder wall, sludge within the gallbladder, a coiled echogenic structure within the gallbladder, multiple liver abscesses, and edematous pancreatitis [7].

Initial therapy for gallbladder ascariasis should involve conservative treatment, unless an associated disease is present or a complication arises [8–11]. Albendazole 400 mg orally administered as a single dose or mebendazole 100 mg twice a day for 3 days or 500 mg as a single dose are both accepted alternatives. Mebendazole is especially suitable for patients when ascariasis coexists with whipworm infection [12, 13]. It is a treatment of choice in our institution and has been proven successful.

Surgical treatment is reserved for cases when spontaneous clearance of worms does not happen and conservative treatment is not successful, as well as when there is dead worm inside the gallbladder and worm associated with calculi. In our experience these are very rare situations and until today, none of our patients required surgery.

## Conclusions

Although rare in occurrence, gallbladder ascariasis should be considered in all patients presenting with abdominal pain, distension, colic, nausea, anorexia, and intermittent diarrhea associated with jaundice, nausea, vomiting, fever, and severe radiating pain. Eosinophilia in blood count, ova and parasites on stool examination, and single, long, linear or curved echogenic structure without acoustic shadowing, looking like an anechogenic tube, and characteristic movement of these long echogenic structures within the bile duct found on abdominal ultrasound are conclusive for diagnosis. Mebendazole is an effective drug for the treatment. Surgical treatment is rarely needed.
